# Chronic Pain in Older Adults with Psychiatric Disorders: the DoCPPA Study Protocol

**DOI:** 10.1192/j.eurpsy.2026.11566

**Published:** 2026-07-31

**Authors:** H. Saint-Martin, I. Rouch, B. Laurent, A. Edjolo, E. Pongan, M. Herrmann, C. Creac’h, K. Pérès, H. Amieva, J.-M. Dorey

**Affiliations:** 1 Department of Aging Psychiatry, Le Vinatier Hospital, Bron; 2 ACTIVE Team, Bordeaux Population Health Center, University of Bordeaux, Bordeaux; 3 Neuropain Team, Lyon Neuroscience Research Center, Lyon; 4 Memory Clinical and Research Center, University Hospital of Saint-Etienne, Saint-Etienne , France

## Abstract

**Introduction:**

**Chronic pain** (CP) and **psychiatric disorders** (PD) are common in older adults, and they both may significantly impact patients’ functioning and quality of life. However, research on the prevalence and impact of CP in people with PD remains limited – especially in older adults – and psychiatric care often neglects somatic comorbidities.

**Objectives:**

The main objective of the DoCPPA study is to determine the **prevalence** and **characteristics of CP** in older adults with PD followed-up in psychiatric services. Our secondary objective is to estimate **associations between CP and various clinical indicators** related to physical, cognitive, and mental health, as well as quality of life.

**Methods:**

**Setting:** Department of Aging Psychiatry of Le Vinatier Hospital Center (France, Bron), inpatient and outpatient psychiatric services.

**Method/Design:** Cross-sectional monocentric study with 430 older patients (65 and over) with PD, under psychiatric care. The inclusion period will be 36 months.

**Measures:**

- **Pain:** STOPNEP; CSI-A; BPI-SF; history of drug and non-drug analgesic treatment; current analgesic prescription
- **Physical health:** hypertension; diabetes; smoking history; hypercholesterolemia; BMI
- **Mental health:** ICD-10 psychiatric diagnoses and age at onset; GAD-7; CES-D; NPI; history of psychiatric hospitalizations and suicidal behaviors; current psychotropic prescription
- **Cognition:** MMSE; 5-word test; FAB; other neuropsychological tests
- **Autonomy level and quality of life** : type of housing; protective measures; human assistance; IADL; SF-12; PSQI; mini-Zarit

**Results:**

The results are expected in 2026, once the inclusion period has ended.

**Conclusions:**

This study aims to contribute to improving care in aging psychiatry, a field where a major challenge lies in the management of multiple chronic conditions. **Perspectives** at different levels :

Scientific : improve **knowledge** of CP in an understudied population

Clinical : improve the **assessment and management** of pain in this specific population

Public health : improve the coordination of care and **optimise** their use of the healthcare system

**Disclosure of Interest:**

None Declared

